# Id1 Interacts and Stabilizes the Epstein-Barr Virus Latent Membrane Protein 1 (LMP1) in Nasopharyngeal Epithelial Cells

**DOI:** 10.1371/journal.pone.0021176

**Published:** 2011-06-20

**Authors:** Pok Man Hau, Chi Man Tsang, Yim Ling Yip, Michael S. Y. Huen, Sai Wah Tsao

**Affiliations:** 1 Department of Anatomy, Li Ka Shing Faculty of Medicine, The University of Hong Kong, Pokfulam Hong Kong Special Administrative Region; 2 Genome Stability Research Laboratory, Department of Anatomy and Centre for Cancer Research, The University of Hong Kong, Pokfulam Hong Kong Special Administrative Region; Karolinska Institutet, Sweden

## Abstract

The EBV-encoded latent membrane protein 1 (LMP1) functions as a constitutive active form of tumor necrosis factor receptor (TNFR) and activates multiple downstream signaling pathways similar to CD40 signaling in a ligand-independent manner. LMP1 expression in EBV-infected cells has been postulated to play an important role in pathogenesis of nasopharyngeal carcinoma. However, variable levels of LMP1 expression were detected in nasopharyngeal carcinoma. At present, the regulation of LMP1 levels in nasopharyngeal carcinoma is poorly understood. Here we show that LMP1 mRNAs are transcribed in an EBV-positive nasopharyngeal carcinoma (NPC) cell line (C666-1) and other EBV-negative nasopharyngeal carcinoma cells stably re-infected with EBV. The protein levels of LMP1 could readily be detected after incubation with proteasome inhibitor, MG132 suggesting that LMP1 protein is rapidly degraded via proteasome-mediated proteolysis. Interestingly, we observed that Id1 overexpression could stabilize LMP1 protein in EBV-infected cells. In contrary, Id1 knockdown significantly reduced LMP1 levels in cells. Co-immunoprecipitation studies revealed that Id1 interacts with LMP1 by binding to the CTAR1 domain of LMP1. N-terminal region of Id1 is required for the interaction with LMP1. Furthermore, binding of Id1 to LMP1 suppressed polyubiquitination of LMP1 and may be involved in stabilization of LMP1 in EBV-infected nasopharyngeal epithelial cells.

## Introduction

Epstein-Barr virus (EBV, also categorized as human herpesvirus type 4) is the first human oncogenic DNA virus isolated from Burkitt's lymphoma capable to transform B cells *in vitro* [1]. EBV was later shown to be a prototype of gamma herpesvirus that infects the majority of population worldwide. After infection, most people carry the virus in their memory B cells in latent stage. EBV infection is associated with specific types of human malignancies, for example Burkitt's lymphoma, Hodgkin's lymphoma, nasopharyngeal carcinoma and gastric carcinoma [2]. The underlying oncogenic mechanisms of EBV are still poorly understood and pre-existing genetic alterations in the infected host cells are believed to be involved.

Examination of the expression profile of EBV genes in EBV-related malignancies and EBV-derived cell lines have defined four major types of EBV latent infection: Latency 0, 1, 2 and 3 each with distinct EBV gene expression. Nasopharyngeal carcinoma are shown to exhibit type II latency infection and the major EBV genes expressed are EBNA1, EBER, LMP1, LMP2A, LMP2B, BARF1 and BARTs. The LMP1 is well-documented to be an important oncoprotein of EBV. It is a transmembrane protein which localizes at cholesterol-rich lipid raft [3], [4]. LMP1 functions as constitutive active form of tumor necrosis factor receptor (TNFR) and activates multiple downstream signaling pathways similar to CD40 signaling mostly via its C-terminal activation domains (CTAR): CTAR1, CTAR2 and CTAR3 [5].

Using nested RT-PCR, more than 90% of nasopharyngeal carcinoma is shown to be positive in LMP1 expression which supports a role of LMP1 in the pathogenesis of nasopharyngeal carcinoma [6]. Intriguingly, LMP1 protein was only detected at low level in NPC tissues and generally absent in EBV-infected nasopharyngeal carcinoma cells [7]. The oncogenic action of LMP1 may play a more important role at early stage of development of nasopharyngeal carcinoma. Presumably, the levels of LMP1 in EBV-infected cells are tightly regulated by host cellular factors. Earlier studies have reported that intracellular signaling events could modulate LMP1 expression. Chen et al reported that STAT3 could upregulate LMP1 transcript through activating the TR, LMP1 ED-L1 and TR promoters [8]. Recently, Johansson et al showed that p38 activation could promote LMP1 expression in lymphoblastoid cell lines (LCL) [9]. Goormachtigh et al found that LMP1 promoter activity was inhibited by NF-kappaB signaling [10]. However, an opposite conclusion was drawn by Demetriades et al showing that NF-kappaB could activate LMP1 pomoter activity [11]. Independently, using DNA affinity purification and chromatin immunoprecipitation assay, Johansson et al showed that the NK-κB p50-p50 homodimers and p65-p50 heterodimers could bind to LMP1 promoter and upregulate LMP1 expression [12]. The discrepancy of the findings has yet to be resolved. LMP1 expression was also shown to be targeted by BART microRNAs and negatively regulated LMP1 expression [13]. LMP1 has also been shown to be target of degradation via ubiquitin-mediated proteasome degradation pathway [14]. At present, very little information is available on the regulation of degradation rate and stability of LMP1 protein levels in cells.

The inhibitor of DNA binding/differentiation (Id), is a family of helix-loop-helix (HLH) proteins described by Robert Benezra in 1990 [15]. These proteins were characterized as HLH proteins lacking the DNA-binding domain. There are four members of Ids in vertebrates, known as Id1, Id2, Id3 and Id4. They were found to play important roles in development and tumorigenesis. Id1 is often found to be overexpressed in cancer cells and contributes to malignant properties of cancer cells including cell proliferation, invasion and angiogenesis [16]. We had previously reported that LMP1 could upregulate Id1 expression in epithelial cells [17] which was confirmed by independent studies [18], [19]. These findings imply Id1 is a downstream effector of LMP1 which supports the oncogenic role of LMP1. Its involvement in the regulation of LMP1 protein in cells has not been examined.

In this study, we had examined the LMP1 protein levels in different EBV-infected cell lines. Consistent with previous reports, the protein levels of LMP1 were low among all EBV-infected cells examined. However, proteasome inhibitor could effectively stabilize LMP1 protein in all EBV-infected cells. Interestingly, we observed that overexpression of Id1 resulted in higher LMP1 protein level in EBV-infected cells. Detail examination revealed that Id1 could interact and stabilize LMP1 by suppressing LMP1 polyubiquitination. We postulate that Id1 overexpression may play a role in modulating LMP1 protein levels in EBV-infected nasopharyngeal carcinoma cells.

## Results

### Detection of LMP1 transcripts in EBV-infected human epithelial cells

**Figure 1:**
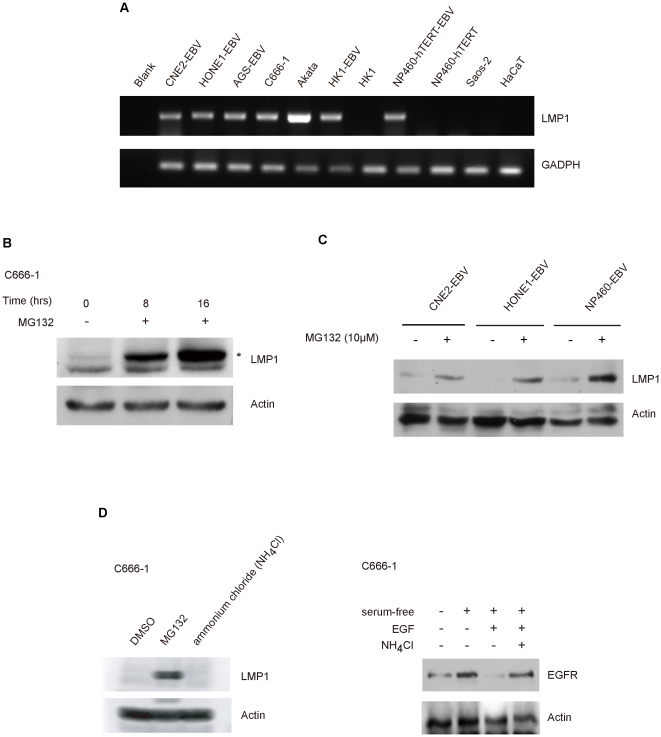
Proteasome inhibitor suppresses LMP1 degradation in EBV-infected epithelial cells. *A*, *LMP1* transcript was detected in EBV-infected cells by RT-PCR. Messenger RNA of LMP1 was detected in EBV-infected cells: HONE1-EBV (undifferentiated NPC cells) and CNE2-EBV (undifferentiated NPC cells), HK1-EBV (differentiated NPC cells), AGS-EBV (gastric adenocarcinoma cells), C666-1 (undifferentiated NPC cells harboring EBV) and NP460-hTERT-EBV (hTERT-immortalized human nasopharyngeal epithelial cells). TPA-treated Akata cells (Burkitt's lymphoma cell line that produces EBV) were used as positive control. HK1 (differentiated NPC cells), NP460-hTERT (hTERT-immortalized human nasopharyngeal epithelial cells), Saos-2 (human osteosarcoma cell line), HaCaT (immortalized human keratinocytes) were used as negative control. The leftmost lane is the cDNA negative control. GADPH was used as loading control. *B*, LMP1 protein could be detected in C666-1 cells after treatment with proteasome inhibitor. Experiments were carried out by treating C666-1 cells with 10 µM MG132 for 8 and 16 hours before analyzed for LMP1 expression by Western blotting. The asterisk indicates the LMP1 specific band. *C*, LMP1 protein accumulated in EBV-infected cells after treatment with proteasome inhibitor. HONE1-EBV, CNE2-EBV, and NP460-hTERT-EBV cells were used in experiment. Experiments were carried out as above except the cells were harvested after 16 hours drug incubation. *D*, LMP1 degradation was inhibited by proteasome inhibitor but not lysosomal inhibitor. (Left panel) C666-1 cells were either treated with 10 µM MG132 or 25 mM ammonium chloride for 16 hours before extracted for Western blotting analysis. (Right panel) C666-1 cells were first serum-starved for 24 hours. Cells were then either treated with PBS or 25 mM ammonium chloride for 30 minutes before stimulated with 2 ng EGF. Cells were harvested 3 hours post-stimulation before extracted for Western blotting analysis.

Previous reports have shown that LMP1 protein levels were either low or undetectable by Western blotting analysis in EBV-infected human epithelial cells in culture [7]. To examine if LMP1 is transcribed in all EBV-infected cells under studies, we extracted total RNA from different EBV-infected cell lines and examined for the presence of *LMP1* transcripts. The *LMP1* transcripts were readily detected in C666-1 which is an EBV-positive nasopharyngeal carcinoma cell line [7], and other cell lines infected with EBV including NP460-hTERT-EBV (hTERT-immortalized nasopharyngeal epithelial cells) [20], HONE1-EBV (nasopharyngeal carcinoma cells), CNE2-EBV (nasopharyngeal carcinoma cells), HK1-EBV (nasopharyngeal carcinoma cells) and AGS-EBV (gastric carcinoma cells). EBV infection of these cell lines were previously established in our laboratories [21], [22], [23] (Fig. 1A). The HONE1 and CNE2 are commonly used NPC cell lines which were initially EBV positive when established from patient biopsies but lost their EBV genomes upon prolonged propagation [24], [25]. The HK1 was isolated from a well-differentiated EBV-negative nasopharyngeal carcinoma [26]. The AGS was isolated from gastric adenocarcinoma [27].

As a positive control for *LMP1* transcription, tetradecanoyl phorbol acetate (TPA) treated-Akata cells were used [28]. LMP1 expression is known to be induced in TPA-treated Akata cells which activate lytic infection of EBV. In consistent with earlier report [7], high level of *LMP1* transcripts was detected in C666-1. The highest level of *LMP1* transcripts was also detected in Akata cells after TPA treatment. The data indicates that *LMP1* is regularly transcribed in all EBV-infected cell lines examined.

### Accumulation of LMP1 in EBV-infected cells treated with proteasome inhibitors

Consistent with previous reports [7], [29], [30], the LMP1 protein levels in C666-1 and the established EBV-infected nasopharyngeal epithelial cell lines were either low or undetectable by Western blotting analysis (Fig. 1B and 1C). Previous study from our laboratory revealed that EBV has established type II latency infection in these EBV-infected cell lines and *LMP1* transcripts were present [31]. We explore if failure to detect LMP1 protein by Western blotting analysis was a result of rapid degradation of LMP1 proteins in EBV-infected epithelial cells. We treated EBV-infected cells with a proteasome inhibitor, MG132, to examine if LMP1 could be accumulated in the treated cells. We observed that LMP1 protein becomes detectable in C666-1 cells by Western blotting after MG132 treatment in a time-dependent manner (Fig. 1B). Furthermore, LMP1 protein could also be detected by Western blotting in other EBV-infected cell lines after treatment with MG132 (Fig. 1C). These results indicated that LMP1 is rapidly degraded in EBV-infected cells despite the active transcription of the *LMP1* gene.

In addition to proteasomal degradation, the LMP1 may also be degraded by lysosomal protein degradation pathway which is commonly involved in the turnover of plasma membrane-bound receptors [32], [33]. LMP1 is a membrane-bound protein which localizes in lipid rafts and may be subjected to lysosomal degradation. We then sought to determine if lysosomal degradation pathway is also involved in LMP1 degradation in EBV-infected cells. C666-1 cells were treated with 25 mM ammonium chloride, which could effectively inhibit the lysosomal degradation pathway as demonstrated in suppressing epithelial growth factor receptor (EGFR) degradation in serum-free C666-1 cells upon epithelial growth factor (EGF) stimulation (Fig. 1D, right panel). We then examined for LMP1 proteinexpression . Unlike treatment with MG132, treatment with ammonium chloride did not result in accumulation of LMP1 protein levels in EBV-infected cells (Fig. 1D, left panel) suggesting that lysosomal degradation pathway may not play a major role in regulation of LMP1 levels in EBV-infected epithelial cells as reported by other group demonstrated similar effects in EBV-infected B cells [14].

### Id1 overexpression increases LMP1 protein levels

**Figure 2:**
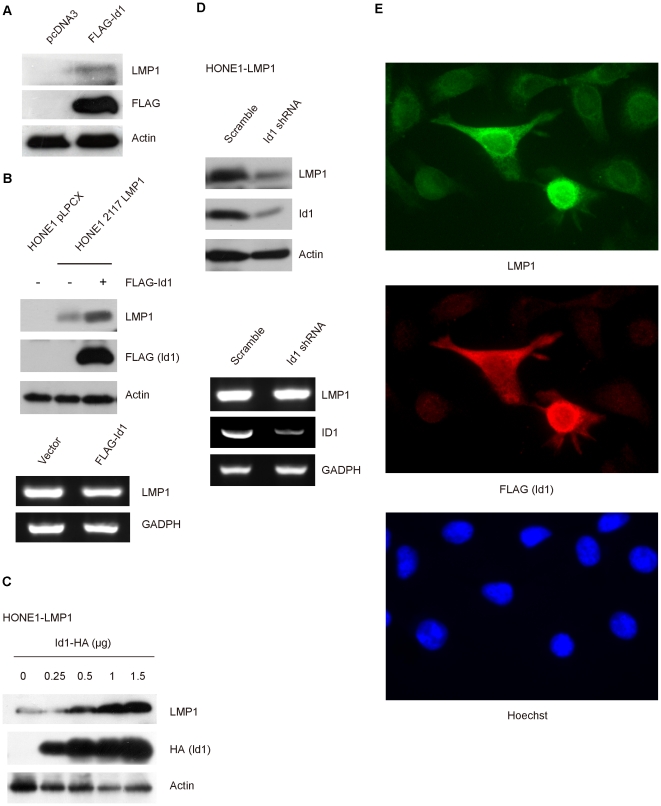
Upregulation of LMP1 level in epithelial cells with Id1 overexpression. *A*, The levels of LMP1 protein could be detected after overexpressing Id1 in C666-1 cells. C666-1 cells were transiently transfected either with vector alone or FLAG-Id1, and were lysed 48 hours post-transfection. Protein were transferred to PVDF membrane and subjected to Western blot analysis with anti-LMP1 and anti-FLAG antibody. *B*, Increase of LMP1 protein levels in HONE1-2117 LMP1 stable cells. HONE1-2117 LMP1 cells were transiently transfected with FLAG-Id1. Cells were examined by Western blot analysis for LMP1 (left panel) and RT-PCR for LMP1 expression with specific primers as described under “Experimental Procedures” (right panel). *C*, Id1 upregulated LMP1 in a dose-dependent manner. Increasing amount of Id1-HA plasmid was transfected into HONE1-2117 LMP1 which stably expressed LMP1. Western blotting was performed 48 hours post transfection. The amount of plasmids used are indicated on the figure in µg. Protein extracts were subjected to Western blot with anti-LMP1 and anti-HA antibody. Actin was used as loading control. *D*. Id1 knockdown was followed by decrease of LMP1 protein level. HONE1-2117 LMP1 was transiently transfected with either scramble shRNA or Id1 specific shRNA. Cells lysates were subjected to Western blotting analysis with anti-LMP1 and anti-Id1 antibody (left panel). RT-PCR was performed with specific primers to detect LMP1 and ID1 gene transcripts. GADPH was used as loading control. *E*. Immunofluorescence examination of HONE1-2117 LMP1 cells transiently transfected with FLAG-Id1. HONE1-2117 LMP1 cells were transfected with FLAG-Id1 plasmid and immunostained with anti-LMP1 (green), anti-FLAG (Id1, Red) antibody and counterstained with Hoechst 33258 to locate the nucleus (see “Experimental Procedures for further details). Higher expression of LMP1 was observed in HONE1-2117 LMP1 cells overexpressing Id1.

Previously, we have reported that LMP1 induces Id1 expression in epithelial cells [17] which was confirmed by other independent studies [18], [19]. Given the functional relationship between LMP1 and Id1, we further examined if Id1 may regulate LMP1 protein levels in cells. Interestingly, we observed that overexpression of Id1 resulted in increase of LMP1 proteins in the EBV-positive C666-1 cells (Fig. 2A) and HONE1 cells re-infected with EBV (Fig. 2B, upper panel). The increase in protein is unlikely to be transcripitonal in nature as RT-PCR examination did not reveal increase in *LMP1* transcript levels in cells subsequent to Id1 overexpression (Fig. 2B, lower panel). Furthermore, transfection of increasing amounts of Id1 plasmid into HONE1 cells stably expressing LMP1 (HONE1-LMP1) revealed progressive increase of LMP1 protein in cells (Fig. 2C). In reverse, knocking down Id1 expression by shRNA in HONE1-LMP1 cells decreased LMP1 protein levels (Fig. 2D, upper panel). RT-PCR examination confirmed that *LMP1* transcript levels were not altered after Id1 knockdown (Fig. 2D, lower panel). Furthermore, immunofluorescence staining of LMP1 in HONE-LMP1 cells reveals that LMP1 expression was higher in cells overexpressing Id1 (Fig. 2E). Taken together, these results suggest that Id1 expression stabilizes LMP1 protein in cells.

### LMP1 interacts with Id1 via the CTAR1 domain

**Figure 3:**
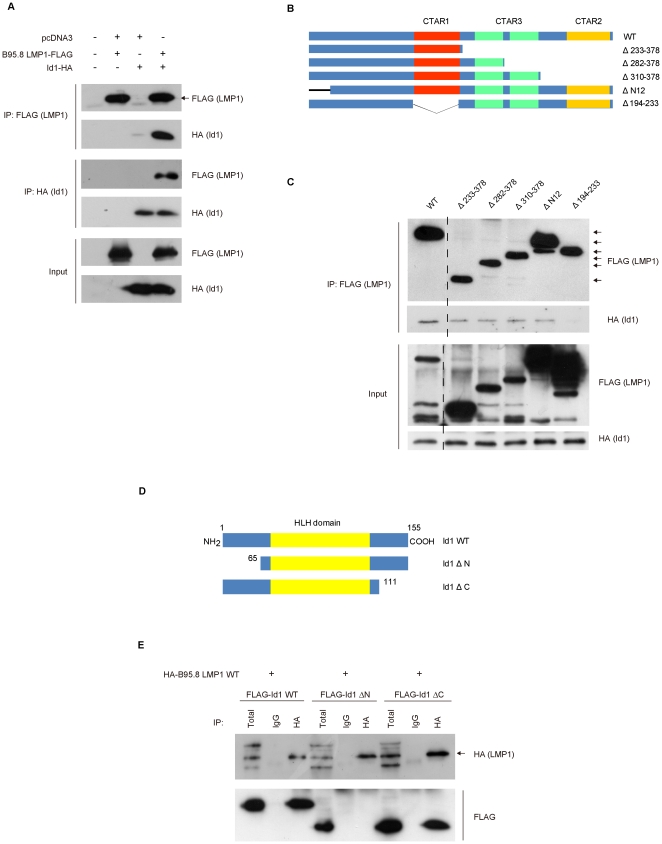
LMP1 interacts with Id1 in cells. *A*, HEK293 cells were transiently transfected with plasmids as shown in the figure. Immunoprecipitation was performed as described in “Experimental Procedures” with either anti-FLAG or anti-HA antibody. The immunoprecipitate was subjected to Western blot analysis with anti-FLAG (M2) and anti-HA (12CA5) antibody. *B*, Schematic illustration of B95.8 LMP1 and LMP1 deletion mutants used in binding experiment. *C*, Immunoprecipitation experiments using FLAG-B95.8 LMP1 and deletion mutants with Id1-HA. HEK293 cells were transfected with constructs encoding the wild type and deletion mutants of LMP1 together with Id1-HA. Immunoprecipitation was performed as described in “Experimental Procedures”, and immunoblotting was performed using indicated antibodies. *D*, Schematic diagram of Id1 and deletion mutants used in binding studies. *E*, Immunoprecipitation experiments using FLAG-Id1 and deletion mutants with HA-B95.8 LMP1 wild type. HEK293 cells were transfected with constructs encoding the wild type and deletion mutants of FLAG-Id1 together with HA-B95.8 LMP1 wild type. Immunoprecipitation was performed as described in “Experimental Procedures.”

We further examined if LMP1 may interact with Id1 by performing binding assay. We observed that LMP1 interacted with Id1 and vice versa (Fig. 3A). LMP1 contains three CTAR domains which mediates most of its downstream signaling pathways. To further define the functional domains of LMP1 which may be involved in the interaction with Id1, we generated LMP1 deletion mutants with truncation of different CTAR domains. As shown in Fig. 3B and 3C, wild type LMP1 and other truncation mutants of LMP1 could all interact with Id1 but not the Δ194–233 mutant (with deleted CTAR1) suggesting that LMP1 interacts with Id1 through the CTAR1 domain.

### Id1 interacts with LMP1 via the N-terminal region

Id1 contains a potential D-box motif (RxxLxxN) at the C-terminus [34], [35], [36], [37] whereas the N-terminal region is important for Id1 interacting with S5A, a subunit of 26S proteasome [38]. We sought to identify the region(s) of Id1 that is important for its interaction with LMP1 using Id1 deletion mutants. FLAG-Id1ΔN harbors a deletion of 1–64 amino acids at the NH_2_ terminus, whereas the FLAG-Id-ΔC lacks 44 amino acids of the COOH-terminus (Fig. 3D). Co-immunoprecipitation experiment revealed that, while both wild type and FLAG-Id1-ΔC bound to LMP1 strongly, FLAG-Id1ΔN could not interact with LMP1 (Fig. 3E) implying that intact N-terminal region of Id1 is essential for Id1-LMP1 interaction.

### Level of LMP1 ubiquitination correlates with the CTAR1 domain integrity

**Figure 4:**
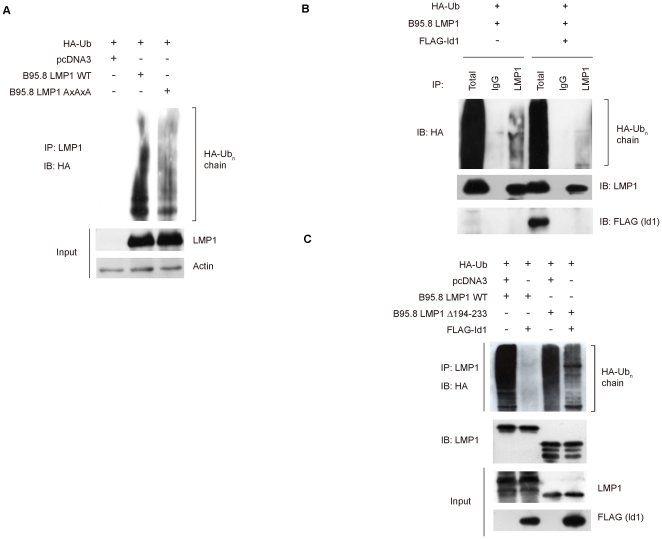
Id1 inhibits LMP1 ubiquitination in epithelial cells. *A*, Ubiquitination status of B95.8 LMP1 wild type and CTARs mutants in epithelial cells. HEK293 cells were transiently transfected with plasmids as the figure shown. *In vivo* ubiquitination assay was performed as described in “Experimental Procedures” MG132 was added and cells were incubated for 6 hours before cell harvest. The extract was immunoprecipitated with mouse monoclonal anti-LMP1 (S12) antibody and subjected to Western blot with anti-HA antibody to detect the polyubiquitin chain of LMP1 and its mutants. *B*, Id1 overexpression reduced LMP1 ubiquitination. HEK293 cells were transiently transfected with plasmids as the figure shown. MG132 was added to the cells and incubated for 6 hours before cell harvest. Immunoprecipitation was performed with anti-FLAG antibody and subjected to Western blot with anti-HA antibody to detect the polyubiquitin chain. *C*, Id1 binds to LMP1 and reduces LMP1 ubiquitination. HEK293 cells were cotransfected either with wild type (WT) B95.8 LMP1 or B95.8 LMP1Δ194–233, with or without FLAG-Id1. Cells extracts were prepared and subjected to immunoprecipitation followed by Western blot analysis as shown in Fig. 4B.

Some hints of the regulation of LMP1 degradation were obtained by an earlier study indicating that LMP1 ubiquitination obeys the N-end rule, i.e. instead of conjugating ubiquitin at internal lysine residue of substrate, the ubiquitin is conjugated at the N-terminus [14]. Mutation of CTAR1 domain resulted in decrease of LMP1 ubiquitination [39]. These two evidences conferred the idea that LMP1 ubiquitination may depend on the integrity of CTAR domains. Based on these studies, we investigated the ubiquitination status of LMP1 with mutations in CTAR1 domain. *In vivo* ubiquitination assay revealed that a lower level of polyubiquination of the CTAR1 mutant (P^204^×Q^206^×T^208^→AxAxA) compared to wild type LMP1 protein (Fig. 4A). Our results support the importance of CTAR1 domain in regulating the LMP1 polyubiquitination.

### Id1 overexpression reduces LMP1 polyubiquitination

To gain insight into how Id1 stabilizes LMP1 protein in cells, we examined if Id1 might interact and stabilize LMP1 by suppressing its ubiquitination. We performed *in vivo* ubiquitination assay in cells cotransfected with wild type LMP1 and Id1 expressing plasmid or control vectors. As shown in Fig. 4B, Id1 overexpression reduced LMP1 ubiquitination significantly. To further clarify the importance of Id1 in reducing LMP1 ubiquitination, we carried out another *in viv*o ubiquitination experiment to check if LMP1-Id1 interaction may affect the degree of LMP1 ubiquitination. Similar to Fig. 4A, polyubiquitination of wild type LMP1 was relatively stronger compared to Δ194–233 LMP1 mutant (CTAR1 deleted mutant). Furthermore, Id1 overexpression suppressed the polyubiquitination of wild type LMP1, but only had mild effects on polyubiquitination for Δ194–233 LMP1 mutant (truncated in CTAR1 domain) (Fig. 4C). Our results support the notion that Id1 interacts with LMP1 through the CTAR1 domain and stabilizes LMP1 by reducing its degree of polyubiquitination.

## Discussion

LMP1 is an oncoprotein of EBV encoded by the BNLF-1 open reading frames. LMP1 expression is commonly detected in nasopharyngeal carcinoma [6]. However, the levels of LMP1 protein detected in NPC specimens are highly variable and heterogeneous [7]. Mechanisms of how the LMP1 protein is regulated in EBV-infected nasopharyngeal carcinoma cells are largely unknown.

Previous studies have reported low levels or absence of protein expression in NPC cells [7], [29], [30]. In this study, we showed that LMP1 transcription could be readily detected by RT-PCR in all EBV-infected epithelial cells but the protein levels were either low or absence in Western blotting analysis (Fig. 1A). The LMP1 protein levels in EBV-infected cells appeared to be tightly regulated by proteasome-mediated proteolysis and could be readily detected by Western blotting after treatment with proteasome inhibitor (Fig. 1B, C). This is in agreement with an earlier study by Aviel et al, which reported that LMP1 undergoes ubiquitin-mediated proteolysis in an EBV-transformed lymphoblastoid cell line stably expressing LMP1 [14]. Treatment with ammonium chloride has minimal effects on LMP1 proteins suggested that lysosome-mediated proteolysis, which is commonly found in degrading transmembrane receptor, may not be a major pathway in the degradation of LMP1 in EBV-infected epithelial cells (Fig. 1D).

Independent studies including us have reported that LMP1 upregulates Id1 in epithelial cells [17], [19], [40]. Biological receptors are known to regulate by complex signaling networks in which feedback loops are commonly involved. Positive and negative feedback loops have been extensively documented in various hormonal receptors like estrogen and androgen receptor [41], [42]. We sought to examine if Id1 could regulate LMP1 expression. We observed that Id1 overexpression could indeed stabilize LMP1 protein levels in C666-1 cells which harbors EBV genome and expresses mRNA of LMP1 (Fig. 2A, B). Furthermore, the increased protein level of LMP1 induced by Id1 is dosage-dependent (Fig. 2C). Conversely, Id1 knockdown results in decrease in LMP1 (Fig. 2D). Taken together, these evidences provide support that Id1 directly stabilize LMP1 through mechanism that is not related to transcription.

We also showed that LMP1 could interact with Id1 in epithelial cells (Fig. 3A). Using deletion mutants, we provided evidences that LMP1 interacts with Id1 through the LMP1 CTAR1 domain (Fig. 3C). We also showed that the N-terminal region of Id1 is required to bind LMP1 (Fig. 3E). Previous study has shown that Id1 interacts with S5A, a subunit of 26S proteasome [38]. The interaction counteracts the inhibition of MyoD and E12 DNA binding ability by Id1 during myogenic differentiation [43]. Several reports indicate that Id1 is targeted by APC/C-Cdh1 complex which leads to mitotic deregulation [34], [35], [36], [37]. Binding studies further reveal that Id1 interacts with S5A via the N-terminal region. Id1 contains a potential D-box motif (RxxLxxN) at the C-terminus. The interaction between Id1 and S5A, a subunit of proteasome; and the requirement of N-terminus of Id1 to interact with LMP1 provide support that LMP1 may be regulated by Id1 which correlates with proteasome-mediated proteolysis.

We have further investigated the degree of ubiquitination of LMP1 CTAR1 mutant when transfected into cells. Earlier findings by Rothenberger et al. showed that the TRAF binding site – CTAR1 was involved in regulating LMP1 protein stability [39]. We repeat the study by comparing the ubiquitination status of wild type LMP1 and CTAR1 point mutant. In agreement with previous study, the CTAR1 mutant is less ubiquitinated compared to wild type LMP1. Therefore, our results reveal that the integrity of CTAR1 domain is important in controlling LMP1 ubiquitination. Using similar approach, we determined that CTAR2 is also involved in determining LMP1 ubiquitination (data not shown). Future experiments are warrant to delineate the mechanism of CTAR2 in controlling LMP1 ubiquitination.

Two pieces of information are crucial in understanding the function of Id1 on LMP1 upregulation: (1) ubiquitin-mediated proteolysis of LMP1 plays important role in controlling overall LMP1 protein level, and (2) interaction between Id1 and LMP1 may influence LMP1 polyubiquitination. Id1 may interact with LMP1 to suppress ubiquitin-mediate proteolysis of LMP1.

LMP1 is targeted to ubiquitin-mediated proteolysis, however, the ubiquitin ligase(s) which is responsible for ubiquitin-conjugation remain unknown. The EBV latent membrane protein, LMP2A, which contains a PY motif at the N-terminal end, associates with HECT family of ubiquitin ligases via the WW domain [44]. The PY motif is however, not present in LMP1. More recently, LMP1 was found to contain one canonical and one crytic HOS motif that can be recognized by SCF^HOS/betaTrCP^ E3 ubiquitin ligase [45]. However, the binding of LMP1 with SCF^HOS/betaTrCP^ was shown to only affect the degree of NF-κB activation but not LMP1 protein stability. Detail regulation of LMP1 protein levels by ubiquitin-mediated proteasome degradation remains to be elucidated.

In summary, our study reveals a new functional link between LMP1 levels and Id1 expression, a downstream effector of LMP1 signaling. Our results revealed a complex interplay of host cellular proteins and EBV encoded products in EBV-infected cells.

## Materials and Methods

### Cell culture and transfection of genes

HEK293T, AGS, Saos-2 and HaCaT cells were obtained from ATCC and were cultured in DMEM and F12K media supplemented with 10% fetal bovine serum (FBS) respectively. HONE1 cells [46], stable EBV-reinfected nasopharyngeal cancer cell lines (HONE1-EBV and CNE2-EBV [25]), EBV-reinfected stomach adenocarcinoma cell line (AGS) and EBV-positive nasopharyngeal carcinoma cells (C666-1) [7] were cultured in RPMI 1640 medium supplemented with 10% FBS [20]. NP460-hTERT cells (Immortalized nasopharyngeal epithelial cells expressing hTERT) [47] and the stable EBV-infected cells were cultured in Defined-KSFM (GIBCO) and Epilife (Gibco) in 1∶1 ratio [47]. All cell lines were maintained in 5% CO_2_ at 37°C. Transfection of genes into cells was performed using Lipofectamine 2000 (Invitrogen) or Fugene HD (Roche) following the manufacturers' protocols.

### Materials

MG132, an inhibitor of proteasome, was obtained from Calbiochem (San Diego, CA). Primary antibodies for Id1 (C-20), β-actin (I-19), HA-probe and Oct-A (D-8) were purchased from Santa Cruz Biotechnology (Santa Cruz, CA). Antibody for FLAG (M2) was purchased from Sigma-Aldrich (Sigma-Aldrich). Antibodies for LMP1 and HA (12CA5) were obtained from Dako (DAKO, Glostrup, Denmark) and Roche (Roche, Germany) respectively. Mouse monoclonal antibody against LMP1 (S12) was a kind gift from Professor Jaap Middeldorp (VU University medical center, Netherlands). Alexa-Fluor 488 and 550 conjugated secondary antibodies were obtained from Invitrogen. HRP-conjugated secondary antibodies were from Cell Signaling Technology.

### Plasmids

The construction and properties of B95.8 LMP1 prototype and its mutants in pSG5 are described previously [17]. FLAG epitope tag Id1 in pcDNA3/Neomycin and HA-epitope tag Ubiquitin in pcDNA3/Zeocin were kind gifts from Dr. Patrick Ling (Queensland University of Technology, Australia) and Ivan Dikic (Goethe University Medical School, Germany) respectively. Id1ΔN-FLAG and Id1ΔC-FLAG in pcDNA3-FLAG were gifts from Karl Münger (Harvard Medical Schoo1, US). Id1 shRNA in pSUPER-Retro-Puro was gift from Joan Massague (Memorial Sloan-Kettering Cancer Center, New York, US). Based on the FLAG epitope tag Id1 in pcDNA3, the C-terminal HA epitope tag Id1 was constructed by PCR amplification using Based on the FLAG epitope tag Id1 in pcDNA3, the C-terminal HA epitope tag Id1 was created was PCR amplification using forward primer 5′-GGATCCAGAAAGTCGCCAGTG-3′ and reverse primer 5′-TCTAGATTACAGGCTGGCATAGTCAGGGACGTCATAAGGATATCAGCGACACAAGATG-3′The PCR product was cut with *Bam*HI-*Xba*I and ligated into pcDNA3.

### Generation of prototype B95.8 LMP1 truncation mutants

Based on the prototype B95.8 LMP1 in pSG5, the FLAG-epitope tag B95.8 LMP1 wild type was created by PCR amplification using forward primer 5′-GGATCCATGGACTACAAAGACGATGACGATAAAATGGAACACGACCTTG-3′ and reverse primer 5′-TTAGATTAGTCATAGTAGCTT-3′; the PCR product was cut with *Bam*HI-*Xba*I and ligated into pcDNA3. Other LMP1 truncation mutants were created based on the FLAG-B95.8 LMP1 wild type in pcDNA3 by PCR amplification using the same forward primer and specific reverse primers:

LMP1Δ233–386: 5′-TCTAGATTAGCAGAGTGGGGGTCCGTC-3′;

*LMP1Δ282–386*:5′-TCTAGATTACTGAGGCAGCGGGTCATGTG-3′;

LMP1Δ310–386:5′-TCTAGATTAGCTATGAGGCAGCGGGTC-3′;

The PCR products were cut with *Bam*HI-*Xba*I and ligated into pcDNA3.

The C-terminal FLAG epitope tag B95.8 LMP1 wild type was created by PCR amplification using forward primer 5′-GGATCCATGGAACACGACCTTG-3′ and reverse primer 5′-TCTAGATTTATCGTCATCGTCTTTGTAGTCGTCATAGTAGCTTAGC-3′.The PCR product was cut with *Bam*HI-*Xba*I and ligatedinto pcDNA3. The ΔN12 LMP1 was created by PCR amplification using forward primer 5′-GGATCCATGCGACGGCCCCCTCGAG-3′and reverse primer 5′-TTAGATTAGTCATAGTAGCTT-3′. To create the LMP1 Δ194–233 truncation mutant, two PCR products were first created by forward primer 5′-GGATCCATGGAACACGACCTTG-3′ and reverse primer 5′-AGAGCAGAGTGGGGGTCCGTCATCACTGTGTCGTTGTCCATG-3′, and forward primer 5′-GACGGACCCCCACTCTGCTCT-3′ and reverse primer 5′-TCTAGATTTATCGTCATCGTCTTTGTAGTCGTCATAGTAGCTTAGC-3′ respectively. The PCR products were mixed and amplified with forward primer 5′-GGATCCATGGAACACGACCTTG-3′ and reverse primer 5′-TCTAGATTTATCGTCATCGTCTTTGTAGTCGTCATAGTAGCTTAGC-3′. The PCR product was cut with *Bam*HI-*Xba*I and ligated into pcDNA3.

### Reverse Transcription PCR (RT-PCR) analysis

RNA was extracted using Trizol (Invitrogen) according to manufacturer's instructions. The cDNA synthesis was performed with the SuperScript ® First-Strand Synthesis System for RT-PCR (Invitrogen, USA), according to the manufacturer's protocol. Four µg of RNA was used in cDNA synthesis unless otherwise stated. Semiquantitative PCR analysis was performed with oligonucleotides as followed:

LMP1: forward primer: 5′-TCCTCCTCTTGGCGCTACTG-3′; reverse primer: 5′-TCATCACTGTGTCGTTGTCC-3′,

Id1: forward primer: 5′-CCGGCAAGACAGCGAGCGGTGCG-3′; reverse primer: 5′-GGCGCTGATCTCG CCGTTGAGGG-3′

GADPH: forward primer: 5′- GCCTCCTGCACCACCAACTG-3′; reverse primer: 5′- GCCTCCTGCACCACCAACTG-3′.

### Western blot analysis

Whole cell lysates were extracted with RIPA buffer supplemented with 1: 100 protease inhibitors (Protease inhibitors cocktail, Sigma-Aldrich). Protein concentration was determined by Bio-Rad DC Protein assay. Protein was boiled in SDS-sample buffer for 5 minutes and electrophoresed on 7.5 to 12.5% polyacrylamide gels, and transferred onto PVDF membrane. Non-specific reactivity was blocked with either 1% BSA or 4% non-fat dry milk in TBST for 1 hour. The membrane was incubated with primary antibodies according to the manufacturers' instructions. The membrane was washed with TBST and incubated with HRP-conjugated secondary antibodies for 1 hour, and washed three times with TBST for 30 minutes. The perioxidase activity was detected by ECL plus solution (Amersham) and exposed to light-sensitive films (GE Healthcare, USA). The films were processed with developer and fixer to get the images (Kodak, USA).

### Immunoprecipitation studies

For the interaction studies of Id1 and LMP1, 400 µg of cell lysates were incubated with primary antibodies for 2 hours. And the immunoprecipitates were recovered by incubating with Protein A agarose beads (Thermos, USA) for 1 hour. The beads were washed three times with lysis buffer and boiled with 2× SDS-sample buffer for 10 minutes before subjecting to electrophoresis.

### In vivo Ubiquitination Assays

Cells were transfected with 0.5 µg of HA-Ubiquitin plasmid and 1 µg of LMP1 plasmid. To accumulate the polyubiquitinated protein, cells were treated with 10 µM MG132 for 6 hours before cell harvesting. Whole cell lysates were extracted with RIPA buffer supplemented with protease inhibitors. 250 µg of cell lysates were incubated with primary antibodies for 2 hours. And the immunoprecipitates were recovered by incubating with Protein A agarose beads (Thermos, USA) for 1 hour. The beads were washed three times with lysis buffer and boiled with 2× SDS-sample buffer for 10 minutes before subjecting to electrophoresis.

### Immunofluorescence studies

Cells were washed in PBS and incubated in 4% paraformaldehyde for 15 minutes. Cells were then permeabilized and blocked in solution with 0.1% triton X-100 and 3% BSA for 30 minutes in room temperature. Cells were washed 1 time with PBS and incubated with primary antibodies for 1 hour. Samples were then washed three times with PBS before incubating with secondary antibodies for 1 hour. Cells were counterstained with Hoechst 33258 to visualize the nuclei. The coverslips were mounted on glass slides with fluorescence mounting medium (Dako, Glostrup, Denmark) and visualized using a Carl Zeiss epi-fluorescence microscope.
